# A nearly-missed peritoneal loose body

**DOI:** 10.3389/fonc.2026.1746145

**Published:** 2026-04-24

**Authors:** Wenbin Fu, Xintian Chen, Qian Liu, Shijie Yao

**Affiliations:** 1 Department of Urology, Tianjin First Central Hospital, Tianjin, China; 2 Department of Clinical Medicine, School of Medicine, Nankai University, Tianjin, China

**Keywords:** adrenal gland, incidental finding, laparoscopy, missed diagnosis, peritoneal loose body

## Abstract

Peritoneal loose bodies (PLBs) are rare lesions that are difficult to diagnose directly, often leading to clinical oversight due to this diagnostic challenge. This paper reports a case of a PLB incidentally discovered during retroperitoneoscopic adrenal cyst excision. Due to cyst calcification and the PLB’s proximity to the cyst, it was nearly missed. It was ultimately completely removed after correlating the surgical specimen with repeated reviews of imaging data.Enhancing the diagnostic proficiency for PLBs facilitates early identification and intervention, thus providing valuable reference for clinical decision-making.

## Introduction

Peritoneal loose bodies (PLBs) are rare lesions, typically asymptomatic and difficult to diagnose directly. This diagnostic dilemma often leads to their being overlooked clinically. PLBs are most commonly discovered incidentally during radiological investigations for other abdominal conditions, during autopsies, or during abdominal surgery performed for other indications. In this case report, we present an intriguing case of an intraperitoneal loose body discovered during adrenal cyst resection.

## Case report

**Figure 1:**
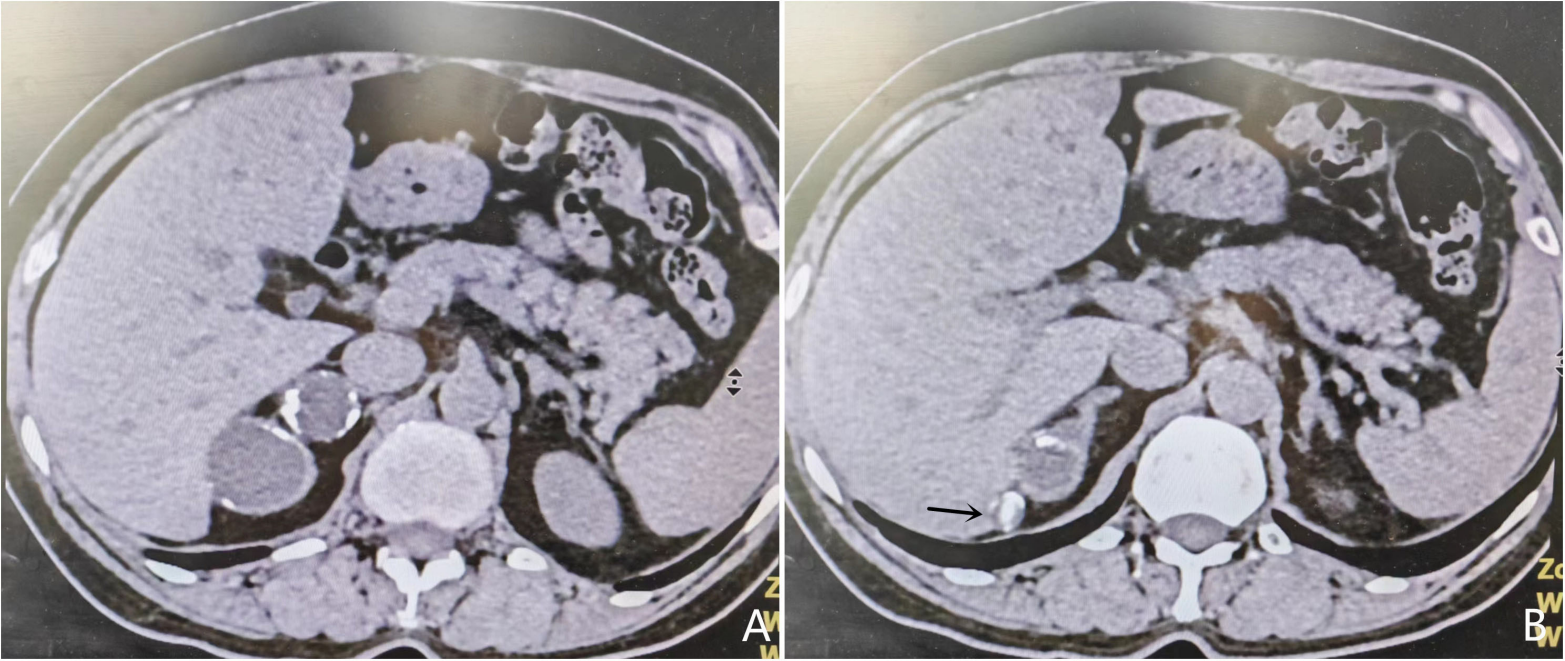
**(A)** Adrenal cystic mass with calcification; **(B)** Intraperitoneal loose body.

**Figure 2:**
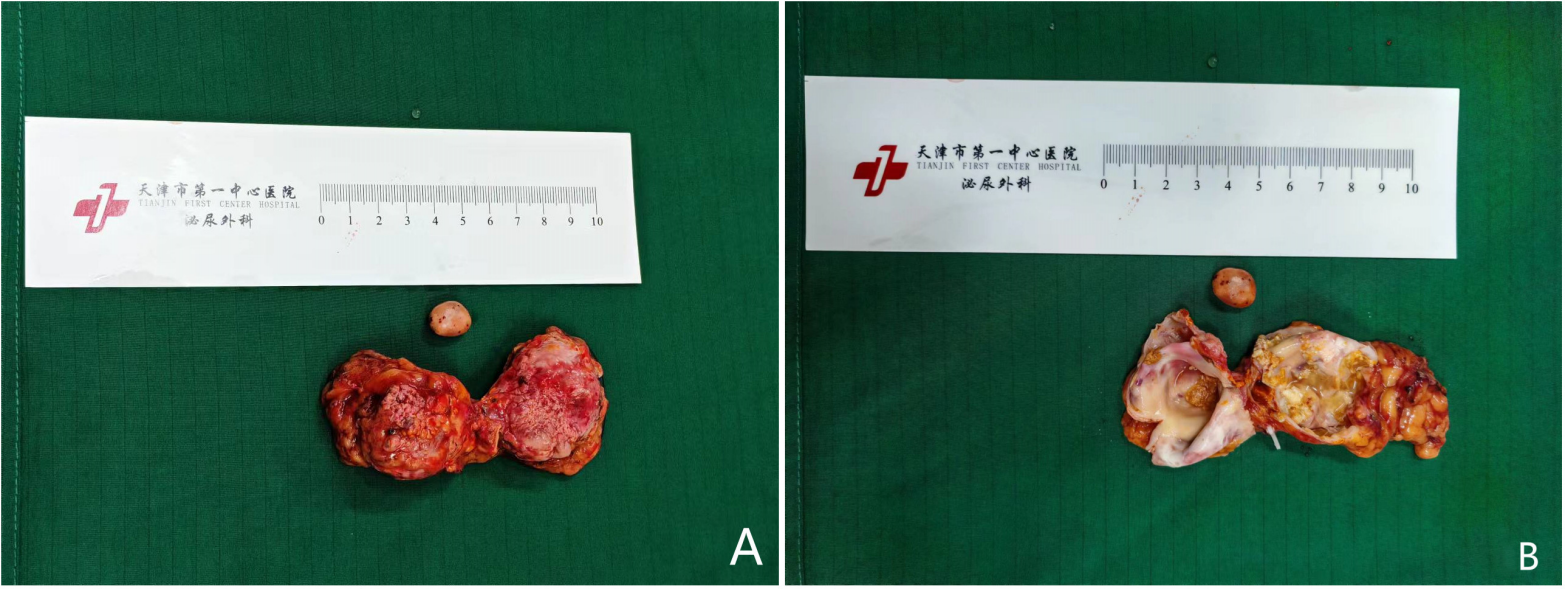
Specimens of the adrenal cystic mass **(A)** and the intraperitoneal loose body **(B)**.

**Figure 3:**
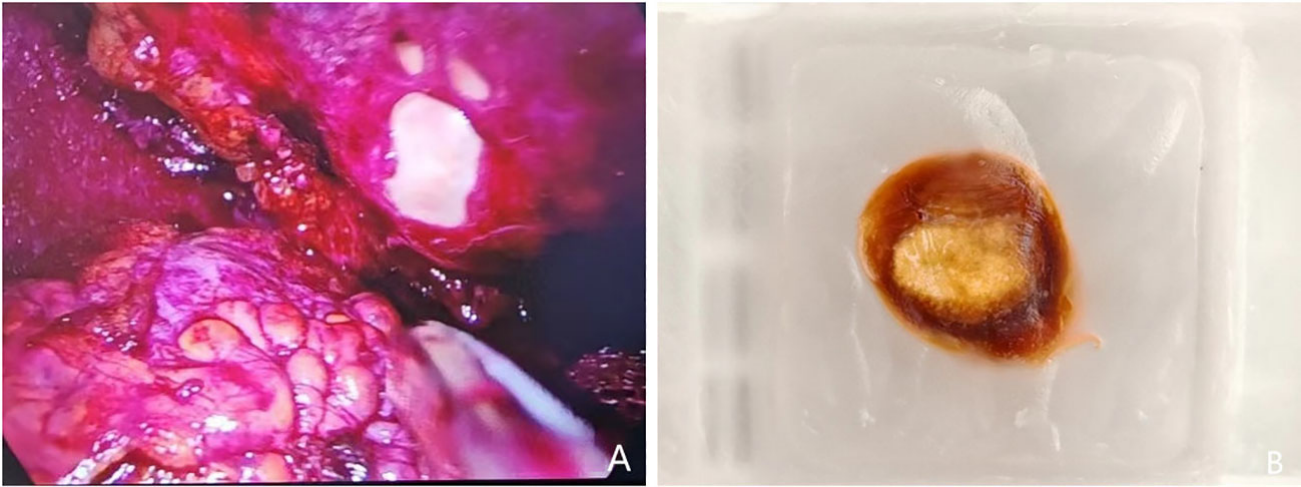
**(A)** Intraperitoneal loose body within the peritoneal rent; **(B)** Paraffin block specimen of the intraperitoneal loose body.

A 52-year-old female patient was admitted on January 24, 2024, with the chief complaint of “A cystic lesion with calcification in the right adrenal gland was identified on computed tomography (CT) one week prior.” She denied symptoms such as hypertension, moon face, or abdominal/lumbar discomfort. Physical examination: Abdomen flat and soft, no tenderness, no costovertebral angle tenderness. Adrenal contrast-enhanced CT report: Multiple cystic lesions with calcification in the right adrenal gland (**Figure 1**). The patient underwent retroperitoneoscopic adrenal cystectomy under general anesthesia. Positioned in the right lateral decubitus position with the lumbar region elevated, a retroperitoneal operative space was established. A laparoscope was inserted. After observation, dissection proceeded along the psoas muscle below the costal margin in the midaxillary line to expose the perirenal fascia. The periadrenal fat tissue was dissected on the ventral and dorsal aspects of the right upper renal pole. Two spherical masses (**Figure 2**), approximately 3-4 cm in diameter, were freed from the adrenal region. The masses were adherent to the peritoneum and carefully dissected free. The central adrenal vein was ligated with a Hem-o-lock clip, and the adrenal cysts were excised.During the surgery, comparison of the excised cystic masses with preoperative imaging revealed a mismatch in shape and size, indicating a missed mass. Multiple careful explorations of the retroperitoneal adrenal region were performed (**Figure 1B**). Ultimately, within a peritoneal rent anterior to the operative field near the liver (**Figure 3**), a date-sized, oval, free stone-like object, approximately 1.3 cm in diameter, was found. After repeated correlation with the surgical specimen and imaging data, it was confirmed as the missing lesion and completely removed.Postoperative pathology: 1. Adrenal simple cyst with calcification. 2. Nodule of fat necrosis, consistent with an intraperitoneal loose body.

## Discussion

Peritoneal loose bodies (PLBs) are rare intraperitoneal lesions, typically diagnosed incidentally during abdominal surgery or autopsy (1). Due to their usual lack of distinct clinical manifestations and limited diagnostic methods, they have long been under-recognized clinically and are easily missed. With advancements in imaging technology in recent years, case reports of PLBs have gradually increased, detection rates have improved, and reported prevalence shows an upward trend. PLBs can occur in patients of all ages but are more common in middle-aged and elderly individuals.Based on diameter, PLBs are classified as: ordinary PLB (< 5 cm), giant PLB (5-10 cm), and super-giant PLB (> 10 cm). PLBs are completely free within the peritoneal cavity and have three key characteristics: (1) High mobility; (2) Absence of blood supply; (3) Negative tumor markers (2). PLBs generally appear as spherical, oval, or irregularly shaped solid masses, pale yellow, grayish-white, or porcelain white in color, with a smooth surface and firm texture.Reports of PLBs causing symptoms are scarce. On one hand, ordinary PLBs usually lack characteristic symptoms. Giant PLBs may cause compressive symptoms in the digestive or urinary systems (e.g., intestinal obstruction, urinary retention, urinary tract infection) due to their large size and mobility, but these symptoms are non-specific. On the other hand, PLBs are extremely rare clinically, and most physicians and radiologists lack awareness of them, leading to frequent missed diagnoses.This case highlights the importance of meticulous review of preoperative and intraoperative imaging. As clinicians, we should not only improve surgical skills but also maintain rigorous clinical thinking to minimize the risk of missed or misdiagnosis (3). CT and MRI can help differentiate PLBs from other lesions. Differential diagnoses include: (1) Benign masses: Leiomyoma, fibroma, visceral organ cysts; (2) Malignant tumors: Colorectal cancer, ovarian cancer, metastases; (3) Calcified lesions: Urinary calculi, gallstones, appendicoliths; (4) Tuberculous granulomas; (5) Other lesions: Calcified lymph nodes, intraperitoneal foreign bodies.The exact etiology of PLBs is unreported. Most research suggests they originate from infarcted, necrotic, and detached epiploic appendages of the omentum, appendix, or intestine. These become completely independent free bodies within the peritoneum. Internal calcification gradually occurs, while the external surface enlarges in a “snowball” fashion by accumulating proteins and exfoliated cells during movement within the peritoneal cavity. Other proposed causes include tissue torsion, peritoneal adhesions, calcification around foreign bodies, or chronic inflammatory hyperplasia due to long-term inflammation (4).

Clinically, PLBs can change position with body movement, peristalsis, or respiration. Larger PLBs have greater mobility, increasing the likelihood of causing recurrent abdominal discomfort and gastrointestinal or urinary symptoms. Therefore, clinicians should enhance their recognition and diagnostic capabilities for PLBs (5). This aids in timely detection, avoiding missed or incorrect diagnoses, and helps select appropriate clinical management strategies:(1)Incidentally discovered ordinary asymptomatic PLB: Follow-up observation may be chosen.(2)PLB discovered unexpectedly during surgery: Should be promptly removed and sent for pathological examination.(3)Giant or super-giant PLB, or PLB located in the gastrointestinal tract, urinary system, or other locations with associated clinical symptoms: Surgical removal is generally recommended to relieve symptoms and prevent complications.

In summary, peritoneal loose bodies (PLBs) are extremely rare lesions with non-specific symptoms, posing challenges for preoperative diagnosis and being highly prone to missed diagnosis (6). Imaging modalities such as CT, ultrasound, and MRI can help assess the size and nature of the mass. Surgical exploration and timely removal of the PLB remain the primary treatment methods.

## Data Availability

The original contributions presented in the study are included in the article/**Supplementary Material**. Further inquiries can be directed to the corresponding author.
